# Platelet activation and aggregation after aneurysmal subarachnoid hemorrhage

**DOI:** 10.1186/s12883-018-1062-z

**Published:** 2018-04-28

**Authors:** Pauline Perez, Anne-Claire Lukaszewicz, Stephanie Lenck, Rémy Nizard, Ludovic Drouet, Didier Payen

**Affiliations:** 1 Anesthesiology and Critical Care Department, Lariboisière Hospital, Paris, France; 2 Department of Neuroradiology, Lariboisière Hospital, Paris, France; 3 Department of Orthopedic Surgery and Traumatology, Lariboisière Hospital, Paris, France; 4 Angio-Hematology Department (L.D.), Lariboisière Hospital, Univ Paris Diderot, Sorbonne Paris Cité, Paris, France; 5 Inserm U1160, Lariboisière Hospital, Univ Paris Diderot, Sorbonne Paris Cité, Paris, France

**Keywords:** Brain, Vasospasm, Inflammation, Coagulation factors, Stroke, Platelet, Cerebral Aneurysm, Subarachnoid hemorrhage, Inflammation, Brain injury

## Abstract

**Background:**

Endovascular techniques have proven beneficial in the treatment of aneurysmal subarachnoid hemorrhage (aSAH), but with high risk of arterial clotting, emboli and dissection. Platelet activation and alterations in hemostasis may contribute to these complications. We investigated platelet activation and aggregation pathways in aSAH patients who underwent endovascular treatment.

**Methods:**

Two blood samples were taken, in the early days after bleeding and during the period at risk of vasospasm. We studied platelet activation through the expression of GpIIbIIIa and P-selectin as well as aggregation rate in the presence of agonists. Platelets from aSAH patients were compared with those from orthopedic postoperative patients (POSTOP).

**Results:**

Platelets in aSAH were initially spontaneously activated and remained so over time. aSAH platelets were further activated with rapid aggregation in the presence of agonists, particularly ADP, with behavior comparable to POSTOP platelets.

**Conclusions:**

aSAH platelets showed prolonged increases in activation and aggregation. Therapies targeting the ADP pathway might reduce the risk of clotting and ischemic events in this context among patients requiring multiple endovascular procedures.

**Trial registration:**

Not applicable.

**Electronic supplementary material:**

The online version of this article (10.1186/s12883-018-1062-z) contains supplementary material, which is available to authorized users.

## Background

Aneurysmal subarachnoid hemorrhage (aSAH) is a severe vascular pathology that is incompletely understood. One of the most disastrous evolutions of aSAH is the occurrence of vasospasm followed often by delayed cerebral ischemia (DCI). Endovascular techniques have proven more beneficial than surgical techniques in treatment of the aneurysm [1] and may also help treat vasospasm. However, such procedures expose patients to high risks of arterial clotting, emboli and dissection [2]. Platelet activation in aSAH patients has received little attention, but some studies have shown important alterations in aggregation. [3–5]. Animal studies have demonstrated increased platelet aggregation, in particular via the expression of platelet surface receptor glycoprotein GpIIbIIIa, as well as a degradation of the anti-adhesive properties of endothelial cells [4, 6]. In this context, platelet alterations might worsen the consequences of vasospasm and affect the risk-benefit balance of invasive procedures.

Currently, no therapeutic strategy targets platelet alterations in aSAH in order to prevent cerebral emboli or vessel narrowing. A Cochrane meta-analysis [7] carried out in 2007 showed that patients who were treated with antiplatelet agents had secondary ischemia less often than patients that received no antiplatelet agent, but the results were not statistically significant. These patients, based on cohorts mostly undergoing surgical treatment of their aneurysms, have a slightly higher risk of bleeding. Another study suggests that for aSAH patients with high-risk features [8], antiplatelet therapy can significantly reduce the rate of peri-procedural emboli without increasing major systemic or intracranial hemorrhages.

In this study, we examined platelet function according to activation pathways in aSAH patients who were likely to require repeated endovascular procedures. Platelet tests were compared to samples from patients undergoing orthopedic surgery with acute postoperative inflammation.

Our main hypothesis was that the injured cerebral tissue could induce the same degree of inflammation-related platelet alterations as found among postoperative patients. Such long-lasting inflammation might durably modify platelet functions, and could be targeted in future aSAH management.

## Methods

### Patients

This study was performed at the tertiary hospital Lariboisiere (Paris, France) with ethical approval (Comité d’éthique de la Société de Réanimation de Langue Française #14–25, CE SRLF 14–25). A written informed consent was obtained from orthopedics patients. Analysis obtained from routine blood samples after the written information of patient in ICU or their next of kin, according to the French regulation of ethics.

Patients were eligible if they were admitted to the intensive care unit with severe aSAH (Fisher grade III or IV [9]), were over 18 years old, and underwent intravascular treatment for an aneurysm. Patients were excluded if they required a craniotomy or craniectomy or had taken an antiplatelet agent within the previous 10 days.

Orthopedic patients (POSTOP) were included during the first 3 days after hip or knee replacement. Patients receiving antiplatelet therapy, steroids or non-steroidal anti-inflammatory drugs were excluded.

### Vasospasm prevention, diagnosis and treatment

All SAH patients received oral nimodipine (60 mg 4 hourly) and thrombosis prophylaxis (enoxaparin). They were monitored twice a day by trans-cranial Doppler in addition to clinical examination. When vasospasm was suspected, a perfusion CT-Scan or arteriography was performed. If confirmed, vasospasm was treated by mechanical (intra-vascular balloon) or chemical (nimodipine or milrinone) angioplasty. When vasospasm exceeded 50%, patients were treated with a continuous milrinone infusion [10].

### Platelet and hemostasis studies

Blood was sampled within 5 days after aSAH onset (early sample), and around day 10 (late sample) when vasospasm becomes evident if present. The nurse in charge of the patient, assisted by the lab technician, sampled the blood at 9:00 in the morning. POSTOP patients had their blood samples taken within 2 days following surgery.

Platelets were tested at baseline and with agonists as follows: 1) platelet activation with expression of glycoproteins alphaII-beta3 (GpIIbIIIa) and P-selectin (CD62P) at baseline and after stimulation by adenosine diphosphate (ADP) or thrombin receptor activated peptide (TRAP), and an index of phosphorylation of vasodilator-stimulated phosphoprotein (VASP) was recorded. Stimulation with TRAP was considered maximum activation. 2) platelet aggregation in the presence of ADP 0.6 μmol/L or arachidonic acid (AA) 0.2 mg/mL.

### Methods and biological measurements

Inflammatory markers were collected, including leukocyte and platelet counts and a measurement of the plasma fibrinogen level (performed via the Clauss method with an automated device; Diagnostica Stago, Asnière, France).

Platelet function was assessed in citrated blood samples and centrifuged at 200*G for 10 min at room temperature to obtain platelet rich plasma (PRP). In PRP, platelet aggregation was investigated by the turbidimetric method on a Chronolog Aggregometer (Beckman Coulter). Briefly, after stirring PRP at 37 °C for 1 min under constant agitation (1000 rpm), aggregation was induced by the addition of ADP (0.6 μM, 1.25 μM, 2.5 μM and 5 μM) and arachidonic acid (0.8 μM and 1.6 μM, Sigma, Lyon, France).

Different monoclonal antibodies directly coupled with fluorochromes were used at saturating concentrations: clone P2, which was directed against the GPIIbIIIa complex (Beckman Coulter, Fullerton, CA), and the monoclonal antibody anti–P-selectin (anti-CD62P, Becton Dickinson). Platelet activation was characterized by the expression of glycoproteins alphaII-beta3 (GpIIbIIIa) and P-selectin (CD62P) as determined using flow cytometry (Becton Dickinson, Mountain View, CA) at baseline and after stimulation by adenosine diphosphate (5 μmol/l ADP (Coulter, Margency, France)) or thrombin receptor activated peptide (25 μmol/l TRAP (Neosystems, Strasbourg, France)) as well as by an index of phosphorylation of vasodilator-stimulated phosphoprotein (VASP).

In 40 μl of diluted PRP was added.

-Either 20 μl of FITC Mouse Anti-Human CD41b Clone HIP2 BD Pharmingen™ Concentration 25 μg/mL.

-Or 10 μl of PE Mouse Anti-Human CD62P Clone AC1.2 BD Pharmingen™ Concentration 1.5 μg/mL.

For assessment on stimulated platelets the same preparation was activated with 2,5 μl ADP (final concentration 5 μmol/l ADP (Coulter, Margency, France)) or 4 μl TRAP (final concentration 25 μmol/l TRAP (Neosystems, Strasbourg, France)). This preparation was analysed through an automated bench top flow cytometer with pre-established template for each of the parameters (BD Facs Canto II).

### Statistics

Quantitative variables were expressed as median (25–75 percentiles: interquartile range). Assumptions of distribution normality and homogeneity of variance were not realized. Consequently, comparisons between groups were done with a non-parametric test (Man-Whitney test). A *P*-value < 0.05 was considered significant.

## Results

Twelve aSAH patients were included in the study. Five required external ventricular drainage for acute occlusive hydrocephalus and six were mechanically ventilated due to alterations in consciousness. Vasospasm was suspected in six patients and subsequently confirmed by angiography in five patients (VS+). All VS+ cases were treated by balloon angioplasty, in addition to chemical angioplasty for two patients. Four VS+ patients received continuous intravenous milrinone (1–1.5 μg/kg/min, a phosphodiesterase-3 inhibitor) because of recurrent vasospasm [10]. Six patients had cerebral ischemia on MRI at 6 weeks, four in relation to angiographic vasospasm and two initially present after embolization. In total, four patients still had a DCI.

Characteristics of the aSAH patients are summarized in Table 1 (and detailed in Additional file 1: Tables S1 and S2). No significant differences were found between patients who went on to develop DCI or those who did not.

**Table 1:** Characteristics of aSAH patients according to DCI

	aSAH (*n* = 12)	DCI (*n* = 4)	No DCI (*n* = 8)
Age, years	56 [47;60]	55 [51;60]	58 [47;70]
Sex, female	9 (70)	4 (100)	5 (62)
WFNS grade	2 [1;5]	1 [1;5]	2 [1,5;4,5]
Fisher III	2 (17)	0	2 (25)
Fisher IV	10 (83)	4	6 (75)
Number of arteriography	2	4	1
GOS	4.5 [4;5]	4 [3;5]	5 [4;5]

*aSAH* aneurysmal subarachnoid hemorrhage, *DCI* delayed cerebral ischemia, *WFNS grade* World Federation of Neurologic Surgeons Grade, *GOS* Glasgow Outcome Scale. The characteristics were expressed as median [25th; 75th percentiles] or as number of patients (%) when adapted

Six POSTOP patients were included with an average age of 65 years. Blood was sampled at postop day 2 [1;3].

Results in platelet tests are summarized in Table 2. For aSAH platelets, GpIIbIIIa and P-selectin expressions were spontaneously high at baseline, as well as response to ADP. Accordingly, the VASP index in aSAH was at the high end of the normal range. In comparison, POSTOP platelets exhibited the same pattern.

**Table 2:** Activation and aggregation tests in aSAH at early days, and in POSTOP patients. The values are presented as median (25th;75th percentiles)

	aSAH		POSTOP		P-value	Standard values
GpIIbIIIa expression (nb of sites)						
Basal GpIIbIIIa	16,618	[14,174;18,246]	18,267	[16,321;19,114]	0.66	NA
ADP GpIIbIIIa	30,702	[29,347;33,832]	29,547	[28,147;32,665]	0.51	NA
TRAP GpIIbIIIa	30,062	[27,375;31,713]	33,381	[30,316;33,504]	0.44	NA
P-selectin expression (% of cells)						
Basal P-selectin	6	[4;8.5]	4.5	[3.2;5.8]	0.65	< 5
ADP P-selectin	82	[62;83]	77	[71;77]	0.48	[60;70]
TRAP P-selectin	90	[89;93]	93.5	[91.5;94.8]	0.14	> 75
Index VASP (%)	84	[72;85]	83	[79;91]		> 50
Aggregation (%)						
ADP aggregation	29	[22;46]	80	[79;90]	0.14	[14;25]
AA aggregation	82	[76;86]	95	[72;101]	0.39	[85;96]

*aSAH* aneurysmal subarachnoid hemorrhage, *ADP* adenosine diphosphate, *AA* arachidonic acid, *POSTOP* postoperative patients, *TRAP* thrombin receptor activated peptide, *VASP* Vasodilator-stimulated phosphoprotein, *NA* not available

Interestingly, platelet aggregation in response to ADP was faster than to AA. The aggregation rate was more elevated in POSTOP than aSAH group. We did not find differences in platelet tests between early and late aSAH blood samples.

**Fig. 1:**
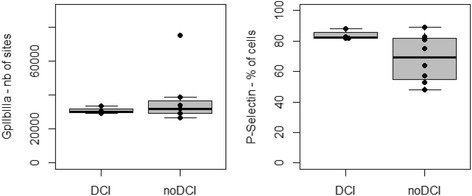
Platelet expressions of GpIIbIIIa and P-selectin trigged by ADP in aSAH patients according to later DCI occurrence (DCI, *n* = 4 and noDCI, *n* = 8). ADP = Adenosine diphosphate, aSAH = aneurysmal Subarachnoid Hemorrhage, DCI = Delayed cerebral ischemia

We analyzed the initial aSAH platelet results according to later DCI occurrence. GpIIbIIIa and P-Selectin expressions were similar for patients who did or did not experience DCI (Fig. 1), as was VASP index (57% [43;71] and 83% [70;86] respectively, *p* = 0.3).

The percentage of aggregated platelets in response to ADP was similarly high in both groups (DCI: 50% [18;83], noDCI: 29% [25;32], *p* = 0.40), while in normal range in response to arachidonic acid (DCI: 91% [66;96], noDCI: 81% [76;82], *p* = 0.29).

## Discussion

Our population of aSAH patients was at high risk of DCI, in relation to their Fisher classifications and the high incidence of vasospasm. In addition to initial aneurysm treatment, patients with vasospasm were exposed to multiple arteriographies when necessary. Cerebral ischemia at 6 weeks was confirmed with MRI for six patients, four related to vasospasm and two to emboli.

The original aim of our study was to focus on aSAH patients treated by endovascular procedures. In SAH patients, platelets were activated especially when triggered by ADP and presented a high aggregability. aSAH induced clear and lasting activation of platelets, similar to the activation measured in POSTOP patients with acute systemic inflammation [11], even when tissue injury was limited. Remarkably, the aggregation rate remained high in the majority of aSAH patients over time, confirming previous work on platelet hyper-aggregability during the vasospasm period [12].

The remarkable response of aSAH platelets to ADP suggests the importance of this pathway and offers a mechanistic hypothesis. Our results resonate with the Cochrane meta-analysis, suggesting the benefit of ticlopidine (a selective inhibitor of P2Y12, ADP pathway receptor) in terms of reduction of DCI and the ineffectiveness of anti-aggregants inhibiting other pathways. Another recent study depicted differences in platelet activation according to clinical severity based on Fisher classification [13], supporting the selection of patients for further studies. Further research is required for patients undergoing endovascular treatment, in order to understand the effects of platelet anti-aggregants on DCI reduction in a context of vasospasm, and on endovascular procedures for dilatation. Unfortunately, the presence of external ventricular drains may limit such treatment in some patients because of the hemorrhagic risk.

The increased expression of P-Selectin may reflect platelet activation after alpha granule release. The relation between receptor expression and platelet exocytosis might also reflect the secretion of serotonin and thromboxane by platelets with their own vasoconstrictive properties.

In our population, milrinone therapy could have dampened the aggregation properties in patients with vasospasm through cAMP phosphodiesterase III isoenzyme inhibition [10, 14]. Milrinone might therefore be the drug of choice in this pathophysiology because of its effects on both platelets and cerebral vasculature during the vasospasm period, after recovery of initial cardiac dysfunction. This hypothesis should be addressed in randomized trial.

Our hypothesis was that aSAH patients would exhibit platelet and hemostasis alterations because of inflammation related to brain injury. The release of damage associated molecular markers from injured brain into the circulation could be a putative mechanism for such systemic inflammation. Accordingly, we chose postoperative orthopedic patients as a control group, with a well-known high risk of thrombosis related to non-septic inflammation after surgery, and considered this a positive control. As expected, in aSAH patients, platelet alterations were equivalent to those seen in the postoperative group. An alternative control group could have been aSAH patients with low Fisher scores, and therefore with low risk of vasospasm and endovascular procedures. However, these patients do not experience the same degree of systemic inflammation and therefore are unlikely to provide a positive control.

The small size of our cohort may have limited the identification of all platelet alterations, especially those associated or unassociated with DCI.

Additionally, the blood samples were not drawn on the same post-bleeding day for all patients, that which may increase the variability of the results. The interpretation of the platelet alterations over the time was also limited by the absence of second late sample in POSTOP patients, who were often discharged.

## Conclusions

Our results confirm the lasting activation and aggregation of platelets in aSAH. Certain therapies, such as milrinone or ticlopidine, could interfere with the ADP activation pathway and reduce the risk of clotting and ischemic events related to endovascular procedures or vasospasm.

## Additional file

Additional file 1: **Table S1.** Biological parameters in aSAH, DCI and no DCI. Biological parameters: platelets count, leukocytes count, fibrinogen, factor VIII, Von Willebrand factor, D-dimer generation in patients with aneurysmal subarachnoid hemorrhage, delayed cerebral ischemia or no delayed cerebral ischemia. **Table S2.** Summary of the characteristics and outcome of each aSAH patient. (DOCX 21 kb)

## Abbreviations

AA: Arachidonic Acid
ADP: Adenosine diphosphate
aSAH: Aneurysmal subarachnoid hemorrhage (aSAH)
CD62P: P-selectin
DCI: Delayed Cerebral Ischemia (DCI)
GpIIbIIIa: Glycoproteins alphaII-beta3
PRP: Platelet rich plasma
TRAP: Thrombin receptor activated peptide
VASP: Vasodilator-stimulated phosphoprotein
VS +: Vasospasm
