# Supplementary material for: Platelet activation and aggregation after aneurysmal subarachnoid hemorrhage
Source: BMC Neurol. 2018 Apr 28;18:57. doi: 10.1186/s12883-018-1062-z (PMC5924502; doi:10.1186/s12883-018-1062-z)
Supplement: Supplementary file 1 — Table S1. Biological parameters in aSAH, DCI and no DCI. Biological parameters: platelets count, leukocytes count, fibrinogen, factor VIII, Von Willebrand factor, D-dimer generation in patients with aneurysmal subarachnoid hemorrhage, delayed cerebral ischemia or no delayed cerebral ischemia. Table S2. Summary of the characteristics and outcome of each aSAH patient. (DOCX 21 kb) [file 12883_2018_1062_MOESM1_ESM.docx]

Online Supplements

Table S1: Biological parameters in aSAH , DCI and no DCI

|  | aSAH | DCI | NoDCI |
| --- | --- | --- | --- |
| Platelets (10^9/L) | 244 [224;368] | 309 [249;369] | 227 [223;302] |
| Leukocytes (10^9/L) | 10.1 [7.8;12.7] | 11.0 [9.65;12.15] | 8.9 [6.7;11.4] |
| Fibrinogen (g/L) | 5.0 [3.9;6.8] | 5.0 [3.9;7.1] | 5.0 [3.9;6.8] |
| FVIII (%) | 300 [210;362] | 205 [164;298] | 184 [173; 220] |
| VWF (%) | 190 [170;235] | 148 [136;209] | 207 [165;258] |
| Gen D-Dimer (AUC) | 67470 | - | - |

Table S2: Summary of the characteristics and outcome of each aSAH patient

| n | Age | Blood sample day | WFNS | Fisher | Localisation of aneurysm | Number of arteriography | Infectious complication | Symptomatic vasospasm | Arteriographic vasospasm | DCI | GOS (6 month) |
| --- | --- | --- | --- | --- | --- | --- | --- | --- | --- | --- | --- |
| 1 | [60 ;70] | 2-7 | I | 4 | ACA | 4 | No | Yes | Yes | Yes | 1 |
| 2 | [60 ;70] | 2-11 | I | 4 | ACA | 3 | Yes | Yes | Yes | Yes | 4 |
| 3 | [40 ;60] | 8-11 | I | 4 | ACA | 4 | Yes | Yes | Yes | Yes | 4 |
| 4 | [20 ;40] | 4-8 | V | 4 | ACA | 1 | No | No | No | No | 4 |
| 5 | [40 ;60] | 6-12 | IV | 4 | ACA | 5 | No | Yes | Yes | Yes | 3 |
| 6 | [40 ;60] | 4-9 | V | 4 | ACA | 1 | No | No | No | No | 5 |
| 7 | [40 ;60] | 4-8 | V | 4 | MCA | 2 | No | Yes | No | Yes | 5 |
| 8 | [40 ;60] | 5-9 | I | 3 | MCA | 1 | Yes | No | No | No | 5 |
| 9 | [60 ;70] | 4-9 | II | 4 | ACA | 1 | Yes | Yes | Yes | Yes | 5 |
| 10 | [40 ;60] | 4-9 | II | 4 | MCA | 1 | No | Yes | No | No | 4 |
| 11 | [60 ;70] | 5-10 | II | 4 | MCA | 1 | No | Yes | No | No | 5 |
| 12 | [40 ;60] | 5-11 | I | 3 | ACA | 1 | no | No | No | No | 5 |

ACA: anterior communicating artery, MCA: middle cerebral artery, DCI: delayed cerebral ischemia, GOS: Glasgow outcome scale
